# Unfairness in Society and Over Time: Understanding Possible Radicalization of People Protesting on Matters of Climate Change

**DOI:** 10.3389/fpsyg.2022.778894

**Published:** 2022-05-27

**Authors:** Amarins Jansma, Kees van den Bos, Beatrice A. de Graaf

**Affiliations:** ^1^Department of Psychology, Utrecht University, Utrecht, Netherlands; ^2^School of Law, Utrecht University, Utrecht, Netherlands; ^3^Department for History and Art History, Utrecht University, Utrecht, Netherlands

**Keywords:** radicalization processes, unfairness, climate protest, social psychology, history, contexts

## Abstract

In this manuscript, we introduce a theoretical model of climate radicalization that integrates social psychological theories of perceived unfairness with historical insights on radicalization to contribute to the knowledge of individuals’ processes of radicalization and non-radicalization in relation to climate change. We define climate radicalization as a process of growing willingness to pursue and/or support radical changes in society that are in conflict with or could pose a threat to the status quo or democratic legal order to reach climate goals. We describe how perceptions of unfairness can play a pivotal role in processes of climate change related radicalization. Without taking any position or judgment regarding climate concerns and associated actions, we suggest that although these behaviors drive many people to participate in peaceful climate protest, they may also lead others to radicalize into breaking the law to achieve their climate goals, possibly in violent ways. This process of climate radicalization, we argue, can be driven by people perceiving certain situations to be blatantly unfair. Specifically, we discuss how radical attitudes and behaviors can be products of perceived unfairness stemming from the past, the future, the immediate social environments of perceivers, as well as those that are spatially distant from them. We further argue that because radicalization processes are shaped by an interaction between individuals and movements, on the one hand, and societal actors and developments, on the other, they tend to develop in non-linear and dynamic ways. We therefore propose that climate radicalization is a (1) dynamic, contingent, and non-linear process, often of an escalating (and sometimes de-escalating) kind, (2) that develops over time, (3) through various interactions between individuals and their contexts, and (4) in which people and groups move back and forth from peaceful protest, through disobedient and unlawful methods, to violent actions. Implications, strengths, and limitations of our model are discussed.

## Introduction

Research suggests a link between people’s perceptions of unfairness and their tendencies to think, feel, and act in radicalizing ways (Van den Bos, 2018, 2020). Following Finkel (2001), we define *perceived unfairness* as “the general feeling that something is not right.” This is typically a very subjective but genuinely felt experience. When individuals notice that certain things are not right (e.g., they feel disadvantaged compared to others or believe the government treats them in an unfair manner), this event can trigger strong feelings and emotions, such as anger, disbelief, and guilt (Barclay et al., 2005; Van den Bos, 2007; Palomäki et al., 2013). Perceived unfairness is often described as an alarming experience because experiencing unfairness threatens people’s sense of who they are and jeopardizes their beliefs of what the world should look like (Van den Bos, 2015). Hence, a confrontation with unfairness may drive extreme thoughts and behaviors, such as rigid worldviews and the violent rejection of democratic principles and the rule of law (Van den Bos, 2018, 2020). This is especially the case when people feel personally uncertain (Van den Bos and Lind, 2009; Hogg et al., 2013) or when they have insufficient capacity to correct self-centered tendencies (Van den Bos and Bal, 2016; Van den Bos, 2018). Whereas extensive research addressed the role of other psychological drivers including social identity and group processes (see McCauley and Moskalenko, 2008; Doosje et al., 2016; Gøtzsche-Astrup et al., 2020), significance quest (see Jasko et al., 2017; Kruglanski et al., 2018), and need for sensation (see Bjørgo, 2011), we focus on unfairness-inspired radicalization processes.

Perceived unfairness has been associated with radicalization of people situated at both ends of the political spectrum (see Moors et al., 2009; Van den Bos et al., 2009; Doosje et al., 2012) and religious groups (see Doosje et al., 2013; De Graaf, 2021; De Graaf and Van den Bos, 2021) both in Western societies and beyond (see Kozloff, 2008; Githens-mazer, 2009; Botha, 2015). A global context in which people currently experience much unfairness is the climate crisis (Della Porta and Parks, 2014; Schlosberg and Collins, 2014; Thomas et al., 2019; Piispa and Kiilakoski, 2021). After all, climate change is linked to a wide range of injustices, from the loss of biodiversity, the extinction of species, to the increase in social inequalities and refugee flows (Intergovernmental Panel on Climate Change [IPCC], 2021). People who notice certain misconduct related to climate issues, may feel outraged about this and decide that immediate action is needed. They may engage in collective action, which refers to any action that individuals take on behalf of a collective organization with the goal of improving the conditions of their own group or another group (Wright et al., 1990). For example, when people find that they themselves, their own group, or other people who matter to them are being denied important goods or rights in society (such as security or public participation), participation in societal protests can become a solution to address this unfairness, a means of effecting social and political change (Folger, 1986; Wright et al., 1990). Participating in protests also benefits the individual, as it provides an opportunity to express grievances that arise from perceived injustice (Gurr, 1970; Berkowitz, 1972; Klandermans, 1997).

Initially, concerned citizens often start with peaceful and legal action to voice their concerns. However, over time some protesters, although clearly not all, may find themselves adhering to more and more radical thoughts, feelings, and behaviors (Van den Bos, 2018). Some may notice that they are not being heard by their governments and therefore decide that disobedient strategies are necessary to gain attention, or that peaceful methods do not bring about much-desired changes quickly enough and therefore consider violence to be a more effective tool. Following Van den Bos (2018), we define *climate radicalization* as “a process of growing willingness to pursue and/or support radical changes in society that are in conflict with or could pose a threat to the status quo or democratic legal order to reach climate goals” (see also Netherlands General Intelligence and Security Service, 2007). To date, it seems that most climate protesters in Western societies stay away from violent repertoires of action. Non-violence is an important value and tactic within the climate movement (de-escalation training is often provided to prevent violent outbursts) (Diprose et al., 2017; Extinction Rebellion, 2019; Bowman and Pickard, 2021). However, climate advocates in Western European countries recently engaged in more and more drastic actions: From locking oneself to fences, disrupting public transport, and blocking the press (BBC News, 2019; Iqbal, 2020), to occupying oil platforms and smashing bank windows to gain attention to climate issues (Carrell, 2019; BBC News, 2021).

**FIGURE 1:**
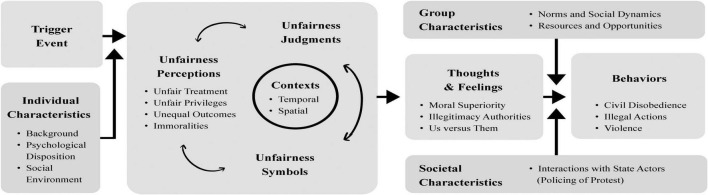
Theoretical model of climate radicalization.

In the present manuscript we propose a theoretical model of climate radicalization. Figure 1 illustrates this model. Our conceptual analysis draws on psychological research on unfairness-inspired radicalization (Van den Bos, 2018, 2020), integrates the sociological concept of injustice frames (Goffman, 1974; Gamson, 1984; Benford and Snow, 2000), and historical case studies of radicalizing movements (Della Porta, 1995; Demant and De Graaf, 2010). Following Goffman (1974), we define the concept of *injustice frames* as “interpretative narratives that help people to perceive, identify and label unjust events within their life space and the world at large.” These frames are generated and adopted by the people that evaluate injustice and may inspire and legitimize their protest activities (Gamson, 1984; Benford and Snow, 2000). Below, we first introduce our model and explain how it can help to understand individuals’ possible radicalization trajectories regarding climate change. After this, we zoom out and address the unfolding of climate radicalization processes of people and groups over time, discussing the role of trigger factors and contingent interactions at the societal level. Finally, we provide comments and future directions concerning our theoretical model and argue for the value of integrating historical insights and concepts, like injustice frames, to the study of psychological radicalization.

## Psychological Processes of Climate Radicalization: The Role of Perceived Unfairness

Today, many people have great concerns about climate change. According to a survey among 1.2 million respondents from 50 countries, 64% of people worldwide believe climate change is an emergency (United Nations Development Programme, 2021). When such concerned individuals become aware of certain misconducts related to climate issues (like observing that the planet is being destroyed by humans), they may form the opinion that this is unfair and experience strong feelings of discomfort (see Van den Bos, 2003). Several types of unfair events can be noted regarding climate change. This may involve inequality of outcomes (those who contribute least to climate change suffer its gravest consequences; Han and Ahn, 2020), instances of unfair treatment (not feeling heard by governments; Piispa and Kiilakoski, 2021), perceived unfair privileges (sustainable living is only for the wealthy; Haugestad et al., 2021), and immoral issues like overconsumption and capitalism (Martiskainen et al., 2020).

Individuals’ background (demographics and life experience), psychological disposition (ideologies, religion, morality, needs, and concerns), and social and national environment (networks, culture, government), determine how they perceive an unfair situation (Van den Bos, 2018; Feddes et al., 2020; De Graaf, 2021). For example, young, female, and highly educated individuals tend to have greater levels of environmental concerns (Jones and Dunlap, 1992; Wahlström et al., 2019) and the same applies to citizens in wealthier or industrialized countries (Kemmelmeier, 2002; Marquart-Pyatt, 2012; Franzen and Vogl, 2013). Such differences in climate change perceptions can explain the different responses of individuals when confronted with climate unfairness. If a young person is very concerned about the climate, then reading the latest IPCC report and seeing that the government is doing nothing in response, will lead quickly to the perception that this is something unfair. Notwithstanding individual differences, a confrontation with unfairness is by many experienced as an alarming event that brings about discomforting feelings and confusion (Van den Bos, 2018).

### Judgments of Unfairness

To understand this alarming experience, people will start looking for meaning since humans have a natural tendency to make sense of what happens to them (Becker, 1971; Heine et al., 2006; Van den Bos and Lind, 2009; Van den Bos and De Graaf, 2020). While interpreting unfairness they will cognitively evaluate important sources in their surroundings, including their interactions with other people in society. Does the state listen to what they have to say with due respect? Are people treated fairly by the police during protests? The experience that someone has treated you in an unfair and unjust way is central to unfairness perceptions (Finkel, 2001; Van den Bos, 2015) and may drive societal protest and the adoption of violent tactics (Klandermans, 1997; Van Stekelenburg and Klandermans, 2013). When protesters are denied the right to demonstrate, are treated differently from other protest groups, or are violently detained, this can fuel a new wave of more hardened or heated forms of protests. Specifically, when people are treated in unfair manners by important people, such as police officers, judges, and politicians, individuals may also begin to distance themselves psychologically from those individuals or institutions conducting the unfair treatment, leading to exclusion from society (Lind and Tyler, 1988; Tyler and Lind, 1992). Thus, through psychological processes of appraisal, people may come to the judgment that something is unfair, which can have far-reaching consequences (both for the individual and for society).

People often make judgments that something is unfair based on specific events or occurrences that are of symbolic value to them. Indeed, in line with theories of symbolic interactionism (Blumer, 1986), specific governmental actions by societal authorities can be important symbols of inhumane conduct for climate protesters. For example, a speech in which a head of state proclaims that climate change is not so bad may lead protesters to conclude that an important authority does not take climate issues seriously and evoke a sense of unfairness. This speech can then become of symbolic value signaling that the way the state treats them is blatantly unfair. Research has shown that such symbols of unfairness can take different forms and include different stimuli such as stickers, posters, and photographs (see Nikolayenko, 2007). Furthermore, these symbols often have to do with historical stories and events (see Githens-Mazer, 2008), or specific actions (or lack thereof) by particular individuals, groups, or institutions (Van den Bos, 2018).

Moreover, when people are constructing fairness judgments, interpreting the event in terms of plausible causes and consequences helps them to make sense of the situation at hand (Benford and Snow, 2000; Della Porta and Parks, 2014). For example, after reading the latest IPCC report and observing large-scale climate impact is being caused by human actions, people may conclude that powerful governments are not fulfilling their societal duties, signing climate agreements but failing to act accordingly, thus endangering the safety of citizens. Protesters may infer that governments are the ones to blame. According to social contract theories (Rousseau, 2004, 2014), people who infer that their government is failing to live up to it commitments may believe that it is justified to withdraw from their own civic duties or, in some cases, that it is the right thing to stop obeying the law and instead take action into their own hands.

### Framing Unfairness and Mobilizing for Change

In our model, unfairness *symbols* refer to salient instances that signal unfairness to individuals perceiving the unfairness and may serve as the basis for their perceptions of unfairness as well as a starting point for protest and processes of radicalization (Van den Bos, 2018, 2020). Through symbols of unfairness people identify solution strategies that guide them in how to respond to the experienced unfairness and act accordingly (Benford and Snow, 2000; Della Porta and Parks, 2014). One way to respond to perceived climate unfairness–at least for individuals living in democratic and open societies–is to demand change through collective forms of action (Wright et al., 1990).

Recent research suggests that perceptions of unfairness are indeed key to collective climate protest (see Della Porta and Parks, 2014; Schlosberg and Collins, 2014; Thomas et al., 2019; Piispa and Kiilakoski, 2021). Notably, in addition to perceived unfairness, there are other social psychological factors that drive people to protest [for an overview see Van Zomeren and Iyer (2009) and Van Stekelenburg and Klandermans (2013), for an integration see Van Zomeren et al. (2008, 2012)]. For example, individuals’ motivation to take climate action can be determined by their level of identification with climate groups (Fritsche et al., 2018; Haugestad et al., 2021), feelings of urgency and responsibility (Basta, 2020; De Moor et al., 2020, 2021) emotional experiences such as anger, fear, guilt, and hope (Kleres and Wettergren, 2017; Martiskainen et al., 2020), and instrumental reasons such as efficacy judgments (Roser-Renouf et al., 2014; Van Zomeren et al., 2019; Wahlström et al., 2019). Yet, in order to map out possible climate radicalization processes, a focus on perceptions of unfairness is particularly important, as perceived unfairness is not only a potential driver of climate protest, but may also drive individuals to break the law and engage in violent behavior.

When individuals use unfairness symbols to understand a certain unfair event, a process of *unfairness framing* takes place, which facilitates what they think, feel, and how they act (Goffman, 1974; Gamson, 1984; Benford and Snow, 2000). An empirical illustration of a unfairness framing is provided by Čapek (1993), who examined a case of environmental contamination in the Carver Terrace community in America (a neighborhood of Texarkana, Texas). The Carver Terrace site was previously used as a waste site for toxic chemical disposal and then became a building ground for residential houses. After several years its new residents came to experience severe health problems. Many other residents were initially unaware of this contamination only until they read about it in the newspaper or were informed by a local environmental organization. According to Čapek, these residents gradually became more aware of the dangerous situation that involved many injustices, including “the poisoning of the land, the neglectful behavior of city and federal authorities, the illnesses and deaths, and the years of hard work lost when property values dropped.” Čapek (1993) Feeling hopeless and distrustful of the conflicting reports presented by outside agencies, a group of residents became more active in protesting to demand justice, eventually ensuring a federal buyout and relocation. Using qualitative in-depth interviews with local residents and other stakeholders, Čapek described how an “environmental justice frame” emerged in this local community as a result of the struggles the residents experienced and how their ability to mobilize for social change was closely tied to picking up this frame.

Perceived unfairness can certainly motivate people to protest peacefully. Furthermore, neutral symbols of information can be important in processes for de-radicalization (see Demant and De Graaf, 2010). In this manuscript, however, we focus on the role of unfairness perceptions leading to possible radicalization into violence. To understand this issue thoroughly, we describe in what follows how unfairness judgments play a role in radicalization processes in different contexts.

### The Temporal Context

Since radical attitudes and behaviors are products of complex interactions between individuals and various changing contexts (Gergen, 1973; Della Porta, 2018; Power and Velez, 2020, 2021; Van den Bos and De Graaf, 2020), it is important to consider that processes of radicalization do not arise and develop in a vacuum, and are only confined to the present. A radicalizing person or movement often has a past history of experiences with unfairness about which they have been indirectly informed through constructed narratives (Van den Bos and De Graaf, 2020). Within groups stories about past unfairness may circulate because they are constantly retold over time.

When people identify with groups that have been wronged in the past, this may be a powerful drive for protest. According to social identity theory (Tajfel and Turner, 1979; Hogg, 2018), this is because when an individual identifies with a group, this is accompanied by a consciousness of similarity, and experiencing shared emotions and fate. It means that a person feels a sense of belonging to a particular group which makes a person more willing to act on behalf of that group (Van Zomeren et al., 2008). Moreover, group identification also explains why protesters may experience negative affect when the group fails (sadness, humiliation), feel positive affect when the group succeeds (happiness, pride), and may experience anger and resentment when faced with a common enemy (McCauley and Moskalenko, 2008). People in the present can retrieve meaning from past instances of unfairness and use them to interpret and react on contemporary experiences with unfairness, possibly in radicalizing ways. Githens-Mazer (2008), for example, shows how injustices that were experienced during the Algerian war in the 1950s and 1960s led to the radicalization of North African Muslims living in Britain several decades later. This is because the stories about past injustices–that were disseminated through symbols, memories, and myths–communicated a history (a series of injustice frames) in which past unfairness against their social group were recognized.

Looking at the climate context of the last decade, past injustices that protesters face may involve specific cases in which they or their group members were wronged, such as violent confrontations with the police (see Baker, 2010; Wahlström, 2011; Diprose et al., 2017; De Moor, 2018). Moreover, we expect protesters’ perceptions to be affected by the occurrence of alleged immoral developments through time, such as national governments’ alleged awareness of the harmful effects of their actions on the planet and its inhabitants (like their role in the fossil industry) already since the 1990s (from then on the IPCC reports were repeatedly published), and their continued denial and evasion of scientists’ warnings (Jäger and Riordan, 1996; Bolin, 2007; Ferns and Amaeshi, 2021).

Importantly, narratives about unfairness in time can focus on the past, but can also point to the future. Expectations of a fair world in the distant future are associated with support and justification for the use of violence in the here and now. By analyzing the speeches of 22 leaders of violent revolutions in the last century, Martin et al. (1990) showed that leaders’ visioning a just future was an important part of the narrative used to legitimize the group’s violence in addressing experienced injustices in the present. Thus, people’s predictions of future unfairness and fairness are pertinent to contemporary perceptions of unfairness and support for revolutionary ideas and violent tactics.

When people stand up against an unfair issue in society such as climate change, they often do so with the goal of reducing or removing that unfairness either in the short run (mitigating ecological disasters already present in the global south) or for the longer haul, that is in the distant future (preventing the prospect of an unhabitable earth). A recent study among Norwegian climate activists (Haugestad et al., 2021), found that a common sentiment and unfairness narrative of young protesters was that they felt they had been cheated of a promised future. The authors explain this experience by pointing to temporal comparisons that young people can make in which the imagination of future consequences of climate change creates feelings of unfairness and frustration, and this legitimizes them to engage in protests (see also Power, 2020). Such stories of future unfairness are rooted in the present, and shape protesters’ contemporary perceptions of unfairness.

Perceptions of climate unfairness are also reflected in notions of intergenerational injustices, the notion that future generations will be left with the climate problems caused by previous generations (Han and Ahn, 2020; Holmberg and Alvinius, 2020). This temporal aspect is important to consider because perceived intergenerational unfairness likely affects the radicalization of different generations differently. The injustice experienced by older generations could be particularly driven by feelings of collective guilt and responsibility, and that of younger generations by fear and hopelessness. Han and Ahn (2020) analyzed the narratives of global youth climate activists and found that younger generations perceive themselves as the victims of climate change and older generations as the villains. In an interview study comparing climate activism in the United Kingdom, Canada, Unites States, and Norway, Martiskainen et al. (2020) found that mothers were motivated to strike for the climate due to altruistic values (on behalf of their children), and young adults because of egoistic values (preserving their own future).

### The Spatial Context

Unfairness judgments are affected by temporal and social comparisons. People often compare their own individual or group situation with what happened in earlier circumstances or with what other individuals or groups receive (Van den Bos et al., 1998). According to theories of relative deprivation (Runciman, 1966; Folger, 1986), feelings of deprivation and frustration arise when social comparisons lead people to conclude that they or their own group are disadvantaged compared to other individuals or groups (such as when a woman receives less pay for the same job compared to a man), are deprived of important rights in society (for example, when the right to demonstrate of some groups is restricted), or receive different treatment (such as when some citizens are more likely to be singled out by law enforcement). For example, when climate protesters observe that their group is violently arrested during protests while other protest groups are escorted by police, this may be judged as something unjust. Perceived relative deprivation can then drive people to participate in social protest (Klandermans, 1997; Power, 2018; Grasso et al., 2019), and move them toward political violence and radicalization (Gurr, 1970; Van den Bos et al., 2009).

Because the impact of climate change is unevenly distributed on a global scale, protesters in Western societies could also derive meaning from unfair experiences of distant others (individuals and groups outside their own social environment, both near and far in geographical space). The idea that people and groups least responsible for climate issues suffer the most severe consequences, despite not being responsible for causing for climate issues, is an important driving force for climate protesters (Han and Ahn, 2020). As such, protesters notice relative deprivation between social groups (the disproportionate burden of environmental hazards placed on less privileged people in terms of socio-economic status, Rainey and Johnson, 2009) and continents (the global South will be first to suffer the burden of climate change, Bond et al., 2020; Piispa and Kiilakoski, 2021). Therefore, social injustices and intercontinental injustices shape protesters’ judgments of unfairness.

Furthermore, the spatial context in which individuals live, both socioeconomically and geographically, affects how they perceive and address the unfairness they perceive. Rather than finding themselves disadvantaged, people protesting in Western societies likely perceive unfair group advantages (see also the literature on relative gratification, Schmitt et al., 2000; Guimond and Dambrun, 2002). For example, an awareness of having privileges in terms of money, knowledge, and safety gained from harmful societal systems (capitalist societies contribute to the climate crisis), may induce feelings of responsibility and guilt for climate change (Wohl et al., 2006; Kleres and Wettergren, 2017). Perceived in-group advantage can then motivate individuals in democratic societies to address unfairness through political action (Iyer et al., 2003; Leach et al., 2006).

Feeling responsible for causing or solving climate issues could motivate individuals in Western societies to come to the aid of marginalized fellow citizens or distant communities living on the other side of the globe. This is especially true for individuals who experience a collective ethic of care and hold strong moral principles such as concern for “the underdog” (Skitka, 2010; Van den Bos, 2018; Bond et al., 2020). Research shows that moral convictions (i.e., strong and absolute beliefs that something is morally right or wrong, Skitka and Mullen, 2002) drive collective action (Van Zomeren et al., 2012; Barth et al., 2015). Notwithstanding the importance of individuals standing up for marginalized groups, strong moral convictions are also linked to a wide range of norm-violations, such as support for and participation in violence (Skitka, 2002; Ginges and Atran, 2009). Additionally, when individuals begin to act morally superior toward others (putting their own moral values before those of others), this can be an indication of radicalization (Van den Bos, 2018, 2020).

### Radicalization of Thoughts, Feelings, and Behaviors

More generally, when people come to understand perceived unfairness they may start radicalizing in their thoughts and feelings (Van den Bos, 2018, 2020). They may rigidly begin to adhere to their own cultural worldviews or political beliefs (e.g., “How I feel about issues is the truth,” see Van Prooijen and Krouwel, 2017), engage in dogmatic us-versus-them thinking (e.g., “The government is our enemy,” see Moghaddam, 2005), start to delegitimize authorities or institutions (e.g., “The police cannot be trusted,” see Sprinzak, 1995, 2009; Saucier et al., 2009), and feel morally superior (e.g., “People who think differently than me are of lesser value,” see Peters, 2005; Täuber and Zomeren, 2012). This process can be reinforced when people feel threatened and uncertain, because then perceived unfairness is more likely to be an alarming experience and connecting with extreme ideas becomes more tempting (Hogg et al., 2013). In the climate context uncertainty feelings play a crucial role: climate change is associated with extinction of species, disappearance of nature, and calls into question the livelihood and safety of all humanity (Intergovernmental Panel on Climate Change [IPCC], 2021). This may trigger intense feelings of fear and despair, shape extreme worldviews, and drive violent action (Van den Bos, 2018, 2020).

It is very difficult to predict when radical thoughts and feelings eventually translate into radical behavior, but there are some important insights that help to understand this issue. When individuals come to reject the rule of law (and associated democratic principles), this is an important turning point in the radicalization process (Van den Bos, 2018). In this phase, processes of delegitimization play an important role. Delegitimization is the psychological process of withdrawing legitimacy, for example from an institution such as a state or from judges in a constitutional democracy (Sprinzak, 1991; Van den Bos, 2020). Through processes of delegitimization, people can distance themselves from societal systems, such as politics, and from principles of democracy and open societies (Popper, 1945). Questioning the legitimacy of a legal system can affect people’s willingness to comply with its laws making engagement in disruptive forms of protest and law-breaking behaviors more likely (Tyler, 2006; Sprinzak, 2009; Jost et al., 2012; Van den Bos, 2018). Thus, the moment climate activists do not feel that they are taken seriously by their government, they may feel disappointed and become distrustful of them. When this happens, there is a change that protesters begin to delegitimize their rules (laws) and executors (police), decide that breaking laws is justified, and consider more extreme approaches against state actors morally justifiable (Van den Bos, 2018). We want to emphasize that many people occasionally oppose certain aspects of the law, but this does not lead them to engage in a violent rejection of the law.

What is noticeable about the climate movement, is that several groups use non-violent civil disobedience as a method of protest (Martiskainen et al., 2020; Thackeray et al., 2020; Furlong and Vignoles, 2021). Civil disobedience can be defined as “the public, intentional, political act in violation of the law, with the purpose of bringing about a change in law or policy” (Rawls, 1971; Chenoweth and Cunningham, 2013). While the vast majority of concerned citizens take peaceful action against climate change, some could go a step further and practice tactics of civil disobedience that push the boundaries of the law leaving a few tempted to resort to violence. For instance, people’s willingness to disobey the law can move far beyond the specific unjust law in question, and spill over into a willingness to flout other unrelated laws as well (Nadler, 2005). It is important to note, however, that although law-breaking is encouraged in this method, the purpose of civil disobedience is to promote democracy (pursuing more just laws) and not to overthrow the democratic system or the rule of law. Without arguing that pushing the boundaries of the law with civil disobedience is necessarily a bad thing [many human rights emerged as a result of this tactic, see also Schuyt’s (1972) analysis of civil disobedience], we must be aware that when people begin to develop contempt for the rule of law and start to sympathize with violent conduct, radicalization into violent extremism might become a realistic possibility (Van den Bos, 2018).

### Group Characteristics and Social Dynamics

At some point, individuals can be attracted to engage in illegal and violent behaviors. Group and societal influences are especially important in this stage (Doosje et al., 2016; Feddes et al., 2020). One of the reasons why this is the case has to do with the observation that groups can provide individuals with radical unfairness frames. To illustrate: someone with sustainable ambitions may first decide to change their own lifestyle and become vegan, eventually realize that this is not enough and become involved in climate protests. After participating in several climate actions, this person may notice that—despite these efforts—their actions are not enough. They may feel outraged by this and start to wonder what could be done about this. Acting on this doubt, a climate movement can present this person with an unfairness frame, identifying the unfairness (the government has been negligent), offering the interpretation (the government only cares about financial interests), and providing an action perspective (join us and together we will demand their responsibilities with disruptive actions).

A group can shape unfairness frames that inspire law-breaking or violent behavior. Movements determine a frame by constructing an unfairness narrative in which they designate the victim of an unfairness (sometimes by amplifying their victimization, see White, 1989; Čapek, 1993). In addition, they also judge who is to blame and this judgment constitutes their action repertoire. When governments, multinationals, or the fossil industry are identified as the primary cause of climate unfairness, they may turn into a common enemy that should be fought against. Such allocations of blame may make illegal or violent protest approaches directed at these actors more justified (Martin et al., 1990; Della Porta, 1995).

Individuals’ actual engagement in radicalizing repertoires of action can furthermore be affected by several group characteristics. Group norms (about breaking the law or using violence) and social dynamics (like social control structures and role models) can determine in what ways individuals will behave during a protest (Bandura, 1977; Bjørgo, 2011). Within groups, individuals have a natural tendency to conform to certain social norms and rules (Perkins and Berkowitz, 1986; Berkowitz, 2010; Gidycz et al., 2011). When the social norm is that violence is justified, then group members are more likely to engage in violence as well (see Littman and Paluck, 2015). On the contrary, when a norm prescribes non-violence, this may prevent people from turning to violent conduct. In addition, social control structures can also constrain certain behaviors (Bandura, 1977). When a group that relies on the principle of non-violence gets intimidated by the police, some individuals may—despite the non-violent group norm—yet intuitively react to such perceived misconduct in an aggressive way. These violent impulses might, however, be corrected by other group members for it signals a deviation from their non-violent group norm. Social dynamics can therefore promote radicalization into violent behavior, but also counteract it.

Furthermore, the resources and opportunities that are available to groups determine their choice for strategies (Della Porta, 2018). For climate protesters in democratic societies, strategies of civil disobedience and law-breaking behavior may be considered an accessible and effective means (see Klein, 2010; Chenoweth and Cunningham, 2013; Engler and Engler, 2016). The use of violence, on the other hand, may backfire because it may lead the general public to view the protest group as less reasonable and reduce their identification with the group (Simpson et al., 2018). This in turn decreases the climate groups’ mobilizing power. Nevertheless opinions on the morality and instrumentality of using violence when protesting for the climate are diverse. Where some people argue that violence could actually be considered a strategically and morally justified tool to groups protesting in oppressed societies (see Bandura, 2002; Vandello et al., 2011), others criticize the dominant adherence to non-violence in the West as well (see Gelderloos, 2007; Malm, 2021).

## The Dynamic Unfolding of Radicalization in Society and Over Time

In the previous sections, we explained how individual trajectories of climate-related radicalization can emerge from experiences of unfairness, how this could then proceed through different feelings, thoughts, and behaviors, and in what ways different temporal, spatial, and social contexts can influence this process. Now we zoom out and discuss several factors that lie outside the individual and which may influence the unfolding of their radicalization processes over time. After all, concrete events often serve as *trigger factors* to start radicalization processes (Feddes et al., 2020). Such trigger factors may concern experiences with discrimination, racism and exclusion, confrontations with authorities, arrests, state scandals, and governmental policies (Feddes et al., 2015, 2020).

It can be quite hard to predict the occurrence of actual trigger events and how they impact processes of radicalization. This is one of reasons why radicalization processes can arise sudden and do not always follow a linear and static trend. Thus, we emphasize that our model should not be interpreted as reflecting gradually developing radicalization processes. Instead, radicalization tends to involve dynamic and non-linear processes over time (see Taylor and Horgan, 2006; Bosi et al., 2014; Della Porta, 2018; Feddes et al., 2020). Several researchers have addressed the dynamic development of radicalization over time [for a social psychological overview, see Feddes et al. (2020); for historical analyses, see English (2008) and De Graaf (2011)]. Furthermore, an important contribution to this line of thought was provided by Della Porta in her work on political violence in the context of social movements (Della Porta, 1995). Through a historical analysis of leftist radicalizing groups in Italy and Germany from the 1960s to the 1990s, Della Porta showed how coincidental interactions between movements and societal actors (states) can suddenly reinforce or slow down radicalization. According to Della Porta, radicalizing individuals and groups often interact with a wide range of societal actors (police, counter-movements, the public) and these interactions can sometimes turn into conflicts.

Perhaps the most important opponent of protesters is the police. Historical case studies of various radicalizing groups show that fierce policing of protests (involving physical confrontations between protesters and police officers) triggered processes of radicalization (see White, 1989; Della Porta, 1995; Della Porta and Reiter, 1998). Police violence often produced an image of an unfair state and this reinforced the unfairness frames protesters adopted. Importantly, these case studies show that through repeated conflicts with the police, protesters who initially pursued non-violence became more willing to use violence over time (at first, only as a defense, later also in an active manner). What is important to note, is that positive interactions between movements and authorities (such as the fair policing of protests) likely reduce or buffer radicalization (Tyler and Jost, 2007; Wahlström, 2007; Tyler, 2011; Baker, 2014).

Conflicts between protesters and police can also occur during climate protests (see Diprose et al., 2017). Especially when climate protesters employ disruptive methods of civil disobedience to reach their climate goals, they will be constantly confronted by police, and their conflicting goals—the police’s job is to enforce laws that protesters are seeking to break—could be a breeding ground for escalation. When climate protesters feel unfairly treated by the police (through intimidation or a violent arrest), this can lead to an escalation of unfairness frames adopted by these protesters which can cause their radicalization to accelerate. Following Della Porta (2018), we therefore believe it is important to keep in mind that although climate protesters currently rarely advocate violence, disruptive forms of protest–that allow for random confrontations between protesters and counterparts–can give way to escalation because violence often emerges from spirals of action and reaction.

In addition, climate protesters may also constantly switch between different action strategies, because they may have multiple unfairness frames at their disposal (Della Porta, 2018). Protesters could start with a peaceful protest (school strike), then move through disobedient and illegal actions (spray-painting walls and joining road blockades), to violent actions (smashing windows) even within a day’s time. They can also shift between violent and non-violent forms of protest or use these strategies simultaneously. Hence, their radicalization process is constantly changing. The dynamic, non-linear, and contingent quality that we assume is underlying many processes of climate radicalization over time is illustrated in Box 1.

**BOX 1. The (hypothetical) unfolding of climate radicalization processes in society and over time.**

In this hypothetical example, concerned citizens in Western societies engage in radicalization processes that are initially triggered by the presence of illegal climate actions in the spatial context of their local neighborhood. People who have read the latest IPCC report may infer important messages from that report that symbolize that their government is blameworthy. Furthermore, historical information in the report reveals that governments should have been aware of climate issues already since the last century and should have started looking for a manner to express the unfairness that this situation triggered. As a result, readers of the report could decide to join an action in which local protesters block a busy traffic road in front of a ministry to gain the government’s attention. Further radicalization could then be influenced by political structures and governmental responses. For example, when protesters believe that politicians are not listening to their concerns and experience little influence through the political and judicial system. In fact, when people experience symbolic events that signal that the government remains negligent in its actions, despite disruptive climate protests reminding them of their responsibilities, citizens may feel that they have no other options but to occupy government buildings in order to be heard. If the state then decides to strictly enforce law and order by arresting these protesters on several occasions, these symbolic events could give way to escalation between protesters and the police, triggering further radicalization. However, when protesters then feel being treated respectfully by the state, because police officers show that they prioritize protesters’ right to demonstrate over public order violations during protests, this symbol of freedom of protest can in fact dampen radicalization processes.

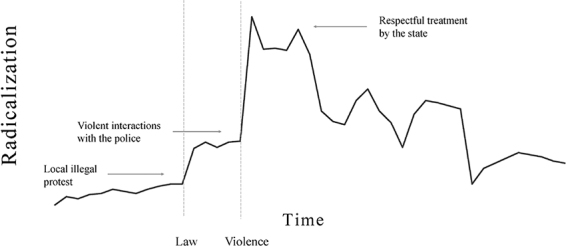

## Summary, Considerations, and Future Directions

In this manuscript we outlined why a contextualized approach to perceived unfairness is pertinent to better understand possible radicalization in relation to climate change. Radical attitudes and actions are, as are all types of behaviors, products of past influences and projected imaginations for the future, and subject to dynamical temporal developments and interactions on a societal level (Gergen, 1973; Della Porta, 1995, 2018; Van den Bos and De Graaf, 2020). Therefore, we propose a balanced approach combining two scientific disciplines that developed relatively in isolation of each other: political history and social psychology. Focusing on macro-level explanations for political violence, historical research has addressed contextual factors, such as the strategies of states, societal structures, and trigger events (Crenshaw, 1981; Della Porta, 1995; De Graaf, 2010). Underlying (cognitive) explanations of the causes of certain instances, however, are often lacking and empirical testing of the outcomes of historical analysis is only rare. Concentrating on micro-level explanations, social psychology linked radicalization to individuals’ motives, cognitive processes and social environment (McCauley and Moskalenko, 2008; Kruglanski et al., 2018; Van den Bos, 2018). Yet the question remains as to how historical processes and societal contexts may affect such radicalization.

To capture this, we introduced a theoretical model of climate radicalization that integrates social psychological theories of unfairness with historical insights. We described how besides individuals’ immediate surroundings, several other contexts, including the past, the future, and those that are spatially distant from them, can play a role in radicalization processes that are driven by perceived unfairness. Drawing on the work of Della Porta (1995, 2018), we then argued that climate radicalization can be seen as a process of (de)escalation that unfolds over time through various interactions between people and their contexts and in which individuals and groups move back and forth from peaceful protest through unlawful methods to violent repertoires of action. Some individuals primarily engage in legal climate protests, and, over time, might start adhering to more radical beliefs, guiding their choices for radial action repertoires. A change in the use of the unfairness frames that people employ may explain why radicalization processes suddenly speed up or slow down. This can be triggered by perceived unfair interactions between people and the state, such as violent confrontations with the police. To better understand if such climate radicalization will occur, it is thus crucial to study what drives individuals to turn to illegal and violent forms of protest, while considering that the development of their radicalization process does not follow a linear and static trend, but rather unfolds in a dynamic, contingent, and non-linear way.

Importantly, a number of issues must be considered when interpreting, developing, and testing our model. First, the model does not present an exclusive representation of people’s radicalization process. Other factors and processes could also be important. For example, previous studies revealed the importance of individuals’ emotions (anger, hate, and contempt), self-corrections (Van den Bos, 2018; Feddes et al., 2020), and quest for significance (Kruglanski et al., 2014). We also propose that sense of urgency could be crucial, because when individuals feel there is no time left this may increase their perceived need for radical actions (Bond et al., 2020). In addition, our model could be tested in other contexts, such as in non-Western samples and societies (see Henrich et al., 2010a,b). The relevance of the model can also be assessed among other forms of radicalization, such as processes of COVID-19 radicalization (see Bartusevičius et al., 2021). Second, although the arrows in our model suggest directional relationships, empirical work must establish such causality. Does perceiving more unfairness lead only to the adoption of radical attitudes, or do people with stronger radical views also perceive more unfairness? In addition, the possibility that different unfairness frames drive different radicalization paths should be explored while considering that people can have multiple frames at their disposal (they often shift between violent and non-violent actions; Della Porta, 2018). Third, we want to remark that although attitudes are often an important predictor of behavior, beliefs not always manifest in behavior, radical actions may also precede attitudes, and, in some cases, radical views remain lacking (with “thrill” seekers, Feddes et al., 2020).

In conclusion, the study of the possible radicalization of climate protest can benefit from insights of historical and societal contexts in which perceptions of unfairness develop over time within individuals and groups participating in these protests. Taking these factors into account is important because radicalization processes do not occur in a contemporary vacuum. Instead, various temporal and spatial contexts inevitably play a role in shaping current perceptions of unfairness that steer radicalization. Furthermore, insights from the field of political and security history have revealed the dynamic course of movement radicalization and its dependence on contingent macro-level interactions. Therefore, radicalization processes may suddenly accelerate (or reverse). Thus, to better understand if, when, and why climate protesters and groups will translate radicalizing attitudes and extreme views into law-breaking or violent behaviors, adopting insights from the field of history is an innovative and promising approach to complement social psychological research on radicalization.

## Author Contributions

AJ wrote the manuscript. KB and BG contributed to, reviewed, and edited the manuscript. All authors planned and discussed the objectives and structure of the manuscript and approved the submitted version.

## Conflict of Interest

The authors declare that the research was conducted in the absence of any commercial or financial relationships that could be construed as a potential conflict of interest.

## Publisher’s Note

All claims expressed in this article are solely those of the authors and do not necessarily represent those of their affiliated organizations, or those of the publisher, the editors and the reviewers. Any product that may be evaluated in this article, or claim that may be made by its manufacturer, is not guaranteed or endorsed by the publisher.
